# Decision-makers’ perspectives on the implementation of COVID-19 self-testing in Mozambique

**DOI:** 10.5588/pha.24.0049

**Published:** 2025-06-04

**Authors:** E. Mavume-Mangunyane, S. Issufo, S. Ndima, E. Valverde, R.R. Peregrino, B. Tasca, C. Penicela, I. Andrade, C. Botão, P.G. Malate, R. Powers, L. Tsope, L. Chimoyi, C. Mulder, I. Spruijt, S. Keller

**Affiliations:** ^1^ Fundação Aurum, Maputo, Mozambique;; ^2^ Faculty of Medicine, University Eduardo Mondlane, Maputo, Mozambique;; ^3^ Vanderbilt University School of Medicine, Nashville, TN, USA;; ^4^ The Aurum Institute, Accra, Ghana;; ^5^ KNCV Tuberculosis Foundation, The Hague, Netherlands;; ^6^ Instituto Nacional de Saúde, Maputo, Mozambique;; ^7^ The Aurum Institute, Johannesburg, South Africa.

**Keywords:** health policy, pandemic response, surveillance

## Abstract

**BACKGROUND:**

To inform future decision-making on pandemic preparedness for COVID-19, we evaluated the acceptability and perceived feasibility of implementation strategies for COVID-19 self-testing among decision-makers in Mozambique. National and provincial directors, heads of programs and division chiefs were selected as decision-makers.

**METHODS:**

We conducted semi-structured interviews with decision-makers involved in COVID-19 diagnosis, management, and policy development. Topics included knowledge and perceptions of COVID-19, testing policies, implementation considerations, and linkage to care. Using a thematic approach, we analysed the interviews.

**RESULTS:**

Seventeen decision-makers were interviewed – most perceived self-testing as an acceptable strategy for early COVID-19 detection. The benefits were improved access to testing, decongesting health facilities, minimising infection risk and decreasing healthcare workers’ workload. Concerns included low testing interest in the post-pandemic period, literacy barriers, affordability and equity issues, mistrust that patients might not take the test due to fear of positive results, and the healthcare system’s capacity to follow up positive cases.

**CONCLUSION:**

COVID-19 self-testing is feasible and acceptable to decision-makers; however, the changing epidemiology has shifted perspectives. This study highlights self-testing’s value in emergencies and pandemic preparedness, enabling rapid detection and isolation of cases, thus minimising the spread of infectious diseases in vulnerable populations in Mozambique and similar contexts.

In March 2020, COVID-19 was declared a state of public calamity at the national level in Mozambique.^1^ Several policies were implemented in the healthcare sector, including an isolation period for confirmed cases, a quarantine period for contacts, prioritisation of health services, expansion of appointment intervals and curfew orders.^2–6^ Despite these measures, the healthcare system was overloaded with the high demand for COVID-19 services during the pandemic’s second and third waves (January and February 2021 and June to August 2021, respectively). As of December 2023, Mozambique’s policies retained the mask obligation for people with respiratory symptoms in health facilities, laboratories, pharmacies, and nursing homes, and they recommend using masks on public transport. General prevention measures such as hand washing, COVID-19 vaccination, social distancing, and cough etiquette continue to be promoted.^7^

Although isolation policies have lifted, seasonal increases in prevalence and occasional smaller outbreaks of COVID-19 can be expected. In line with the WHO Declaration (2023), preparedness for the new influx of cases,^8^ and readiness to implement infection prevention and control measures are essential. A major infection control measure is the early identification and isolation of persons with COVID-19. In 2022, the WHO global survey implemented in 101 countries (31 high-income and 70 low-middle-income countries) reported the existence of a policy supporting COVID-19 self-testing for a range of use cases, including symptomatic and asymptomatic populations.^9^

In Mozambique, the COVID-19-related guidance on rapid testing launched in October 2020 aimed at increasing access to COVID-19 testing through professionally used and facility-based antigen rapid diagnostic tests (Ag-RDTs).^10^ The National Institute of Health also created diagnostic points at hotspots in Maputo City to increase accessibility to COVID-19 testing. Recent studies conducted in low- middle-income countries (LMICs) showed that COVID-19 self-testing using Ag-RDTs is an acceptable strategy for the early identification of persons with COVID-19 among the general public, healthcare workers and decision-makers.^11–14^ Furthermore, other studies have also shown the feasibility of COVID-19 self-testing among persons with COVID-19 symptoms presenting to health facilities.^11,15^ Despite this evidence, no guidance has been developed for COVID-19 self-testing in Mozambique. To inform future decision-making on pandemic preparedness for COVID-19, we evaluated the acceptability, perceived feasibility, and potential implementation strategies for COVID-19 self-testing among decision-makers (DMs) in Mozambique. We defined feasibility as the extent to which COVID-19 self-testing can be successfully implemented within the study setting and acceptability as the extent to which COVID-19 self-testing is judged as suitable, satisfying and attractive to the population of interest: decision-makers.^16^

## METHODS

### Study setting and population

We conducted a cross-sectional qualitative study using in-person and online semi-structured, in-depth interviews (IDIs) between July and September 2023. We conducted a landscape analysis to identify health decision-makers from the Ministry of Health, the National Institute of Health and stakeholders who influence or are involved in developing national health policies. National and provincial directors, heads of programs and chiefs of divisions were selected as decision-makers. A purposeful sampling approach that considered sex, geographical origin, and professional and institutional sampling was used. We invited decision-makers for interviews by email and/or phone. All interviews were organised in a private room in each participant’s workplace.

### Data collection

An interview guide was created that encompassed the themes of knowledge and perceptions of COVID-19 in general**,** acceptability of COVID-19 self-testing, current COVID-19 testing policies, implementation of COVID-19 self-testing and linkage to care following a positive self-test (Supplementary Data 1). The interview guide was developed in English and translated into Portuguese. A team of four researchers (EMM, SI, PGM and CB), including three physicians (female) and one medical anthropologist (male), all with experience in qualitative data collection methods, conducted the interviews in pairs. A total of 17 interviews were conducted (one online and 16 face-to-face). All participants were provided with detailed study information before giving their written informed consent. After being informed according to the informed consent process, all interviewees provided written consent for the interview, audio recording, and data usage for analysis before the commencement of the interview. Interviewees invited were assigned a unique study number for confidentiality. Demographic data were collected at the start of the interview (Table).

**TABLE.** Participants demographic characteristics.

	*n* (%)
Interviews, *n*	17
Age, years, mean (range)	40 (32–61)
Sex	
Female	13 (76)
Male	4 (24)
Level of education	
Higher education	17 (100)
Health system level	
National	10 (59)
Provincial	7 (41)
Level of decision	
National and Provincial Director	8 (47)
Head of Department	9 (53)
Division Chief	6 (35)

### Data analysis

Interviews were audio-recorded and stored in a secure location. Following the recording, interviews were summarised in an interview report and transcribed verbatim. Interviews continued until saturation, and no new themes emerged. The transcripts were entered in NVivo 12^17^ and coded by BT and SN in Portuguese, using both inductive and deductive methods. The research team familiarised themselves with the transcripts and developed a primary codebook based on the interview topic guides (deductive), with new codes added as new themes emerged (inductive). Findings were summarised using a thematic approach guided by the principles of Braun and Clarke^18^ and supporting quotes were identified. The Consolidated criteria for Reporting Qualitative research (COREQ) guidelines^19^ for qualitative research were followed where applicable.

### Ethics statement

The study was approved by the CERC-WHO (CERC.0134 - 18/05/2023), the Institutional Bioethics Committee of the National Institute of Health in Mozambique (222/CIE-INS/2022-28/11/2022), and the National Bioethics Committee of Mozambique (169/CNBS/2022 - 09/03/2023). This study received additional approval from the National Drugs Regulatory Authority (ANARME) to authorise the importation and use of self-test devices in research (48/380/ANARME/2023 - 11/05/2023).

## RESULTS

Seventeen interviews were conducted with decision-makers, predominantly female (13 out of 17 participants). Most decision-makers represented the National Level of the Health System (10 out of 17) and held senior roles, with the majority serving as Heads of Departments (Table). From the analysed interviews, it was evident that all DMs showed a high level of scientific knowledge about COVID-19. Most of them worked or were involved in handling or managing cases related to COVID-19 or other related diseases, thus providing detailed insights into the feasibility and acceptability of implementing COVID-19 self-tests.

### Acceptability of COVID-19 self-testing

Most DMs interviewed perceived self-testing to be acceptable. They reported that this testing modality could substantially benefit decongesting health facilities and decreasing HCWs’ workloads.

It reduces the workload of healthcare personnel; the individual does not have to be moving and exposing himself to other intra-hospital infections or other infections. (Decision-maker, MCC-007).

Additionally, DMs highlighted that the public health benefit of COVID-19 self-testing lies in preventing transmission through the rapid screening of persons with COVID-19 and their contacts and its ability to reduce time to diagnosis, providing rapid results to individuals to take appropriate on-time care.

It plays an important role because as soon as symptoms begin, the person can have a quick test and do it, instead of waiting for an appointment at the hospital, at the laboratory, so it is faster with a self-test than going for a conventional test. Also, self-testing can be offered right at the time of screening, and the patient’s waiting time can be reduced. (Decision-maker, MPC-010).

As perceived by the DMs, self-testing provides an opportunity to increase access to care, benefiting both the individual and the health sector.

No doubt, because in the population we have various social levels, we have levels of the population with access to health services, but the majority of it [population] has difficulty accessing health services, so having self-testing available will undoubtedly be an added value for the health sector. (Decision-maker, MCC-008).

Decision-makers highlighted potential barriers to accepting COVID-19 self-testing among the general population. While describing such barriers, the DMs often provided suggestions for alleviating them. One major barrier was the perceived lack of urgency and importance for testing for COVID-19 among the general public now (September 2023), which has no longer been declared a public health emergency.

Now, in this context that we are already calm, I don’t think it would have much of an impact because people don’t even go there [health facility] much anymore, but in high season, if we have an outbreak again or we have a pandemic here, I think it will be good. (Decision-maker, MCP 014)

DMs described the importance of awareness creation using the media, door-to-door campaigns in health facilities and ensuring easy access to self-testing to enhance knowledge about the availability and purpose of the test. It is seen as fundamental for future implementation of COVID-19 self-testing.

Firstly, I think we need to have a very well-designed communication strategy and implement it at all levels, so we will ensure that people understand what self-testing is about, at the risk of putting a product on the market that people don’t have. A clear understanding of how and what it is for. (Decision-maker, MPC-002).

Our respondents reported that awareness campaigns should also guide individuals on where to seek assistance if they have a positive result.

Mozambicans are not used to testing at home, and even the acceptability of the HIV test is still very timid… if it is positive, what should you do? I’m going to panic. So, there is this psychological part that is very deep. (Decision-maker, MCC-012)

Self-test availability was identified as a condition for implementation. DMs perceived that unless self-tests become widely and readily accessible, the general public would unlikely buy or utilise them. Furthermore, some DMs indicated that to increase acceptability among the general public, COVID-19 self-tests should be made available at low costs or free of charge, particularly in rural areas where purchasing power is very low.

We can’t talk about those of us who have a stable financial condition; we have to see others, so for others, accessibility and price must be very important, especially for people who are in the countryside. (Decision-maker, MCC-005)

Some decision-makers expressed concern about follow-up actions after a positive test result. They have reported that individuals with a positive COVID-19 self-test result might lack the motivation to go to a health facility due to convenience-related factors such as the long waiting times to get assistance in the health facility. On the other hand, there was concern about managing the flow of referred individuals who self-test at home and go directly to a health facility. If self-testing creates a high demand at the health facilities, it could create a potential strain on the health system.

… one thing is to test in the cabinet and get the result, prescribe the treatment, another thing is to receive referred patients from the community; this has always been a challenge; the user is not motivated to wait in a queue. The first point is that it will be a community reference for the health facility, so it is necessary to analyse the flow. (Decision-maker, MPC-009)

### Feasibility of implementing COVID-19 self-testing

Some DMs expressed their concerns about the ability of the public or some sub-populations, such as illiterate individuals, to conduct the COVID-19 self-test accurately, particularly the sample collection. They worried that due to inaccurate self-test execution, the test results would be unreliable and confirmatory testing would be needed in a considerable proportion of the population. To (partly) overcome this barrier, some DMs expressed the importance of providing simplified information on using the COVID-19 self-test.

The main barrier is the collection itself; it is much easier to collect a sample from the oral mucosa, in the mouth, under the tongue than a swab in the nose, so this can be a barrier to the quality of this test, we don’t know if we will have false negatives or if they really are negative... (Decision-maker, MPC-010)

The use of digital health tools was seen as a mechanism that could boost the feasibility of implementing COVID-19 self-testing. According to decision-makers, digital tools function as an element that facilitates the linkage of patients, particularly those who tested positive, to the health facility. They function as an effective and efficient means of supporting the patient referral system. They also reported that integrating self-testing information with the already existing systems, such as the SIS-MA (MOH’s Health Information System for Monitoring and Evaluation), could be key to identifying people who did the test, knowing their results and facilitating the referral process.

So, I think about a tool like this [SIS-MA] that also guarantees continuous reporting. For example, if an individual gets a negative or positive result and he/she is linked to healthcare it is easy for him to continue with the follow-up. Otherwise, he takes the self-test, and when he arrives at the health facility, they [HCWs] don’t recognise or accept the result, and there is a barrier right from the start; he has to do the same thing again, he has to do a huge queue. So, the person won’t see an advantage in self-testing, that’s it. (Decision-maker, MPC-001).

The participants explained that there was a need for the development of an effective and acceptable COVID-19 testing policy with the involvement of all relevant sectors, including the National Directorate of Public Health, National Directorate of Medical Assistance, National Institute of Health, Central of Medicines and Medical Articles, National Drug Regulatory Authority. They highlighted that experiences accumulated during the pandemic should inform the updated guidelines. Some DMs state that regardless of the design of any strategy or guidelines, there must be political will from those making decisions to implement self-testing.

What would make this difficult first is political will. Like everything here, if people understand that this is, in fact, important, as long as there is political will, everything happens, because with political will, even if we don’t have financial means, donors will support. (Decision-maker, MCC-005).

## DISCUSSION

Our study assessed the reflections of decision-makers on the implementation of COVID-19 self-testing. Overall, the findings were consistent with the global context, with decision-makers in Mozambique perceiving COVID-19 self-testing as feasible and acceptable.^13,14,20^ However, there were some potential barriers and facilitators to the uptake of this model of testing. Additionally, DMs recommended creating tailored health promotional messages to expand the reach and uptake of COVID-19 self-testing. There was a consensus among the respondents on the potential of COVID-19 self-testing to alleviate strain on healthcare facilities and reduce the workload of healthcare personnel, benefiting the individuals and the health sector by accelerating the time to diagnosis and appropriate care. This finding is consistent with existing literature on values and preferences of COVID-19 testing in low- and middle-income countries, where HCWs recommend self-testing to scale up COVID-19 testing and increase linkage to COVID-19 treatment.^13^ Additionally, DMs highlighted the potential of the self-test to reduce the burden on health facilities, reduce the workload of HCWs due to the pandemic, and help inform self-isolation and contact investigation, thereby reducing COVID-19 transmission.^21^

Facilitators of the uptake of COVID-19 self-testing were identified, including the availability of the self-tests, removal of facility-based testing barriers and prevention of transmission. These findings are supported by a previous study conducted in Mozambique, which highlights similar facilitators of self-testing for other diseases, such as HIV.^22^ Utilising digital health tools can further facilitate this implementation by integrating self-testing information with existing systems like SIS-MA (Health Information System, Monitoring and Evaluation). A study from Kenya suggested that with the use of digital tools, health services can be delivered effectively and maximise access to health facilities,^23^ aligning with recommendations from the DELPHI study, which advocates for increased investments in digital health infrastructure, adapting user interfaces and experience to expand access, especially for vulnerable groups, and leveraging implementation science to determine which digital health solutions can be quickly scaled up in the country.^24^

DMs also emphasised the importance of leveraging existing communication structures to raise awareness of COVID-19 and self-testing, addressing barriers such as costs, test accuracy and referral processes. This aligns with similar studies implemented among various populations where the informants indicated that a sufficient health workforce must accompany policies and regulations to counsel the public on self-tests and mass awareness campaigns to dispel myths around COVID-19.^15,21,22,25,26^ Based on our respondents and other studies regarding barriers to self-testing, it is believed that providing easily accessible, affordable, or free self-testing would be an essential strategy for those who cannot afford it. This finding corroborates existing literature on the uptake of COVID-19 self-testing in Durban, Kenya and Indonesia,^13,21,27,28^ where informants suggested that self-tests must be cheap or provided free of charge. In one of the literature from Kenya, participants suggested it should be approximately the same as a pregnancy test (USD 2),^20^ and in Indonesia, they suggested it be subsidised.^13^

Regarding the ability of the public to accurately conduct the COVID-19 self-test, the availability of simplified information on how to use the COVID-19 self-test can respond to the concern about the impact of the reliability of the self-test. This concern is mirrored in studies from Indonesia and Nigeria, where some informants perceived the possibility of user error in self-testing, suggesting education as a key strategy.^13,14^

Addressing the follow-up process for people with positive results in COVID-19 self-testing and managing the potential strain on the health system due to a high influx of referred patients is crucial to ensure self-testing feasibility in Mozambique. Digital health tools can potentially overcome this barrier by facilitating the linkage to care. This concern was also found in other studies in LMICs and other contexts, where the use of educational platforms and trained personnel to provide counselling before and after the use of self-tests was suggested as a possible solution.^14,21,22,28,29^ When considering conditions for future implementation, the DMs highlighted the need to develop an effective and acceptable COVID-19 testing policy with the involvement of all relevant sectors. In humanitarian crises, like the ongoing conflict in Cabo Delgado Province, northern Mozambique,^30^ or in the case of cyclones like IDAI, Kenneth and Freddy,^31,32^ hospitals are destroyed, medical personnel are displaced, and the provision of medical supplies, including personal protective equipment to prevent the transmission of infectious disease is inadequate, making the refugees and internally displaced persons (IDPs) susceptible to outbreaks. The lessons learned during the pandemic should inform the update of guidelines. Some DMs stated that regardless of the design of any strategy or guidelines, there must be political will, and it is one of the determinants for the successful implementation of COVID-19 self-testing in Mozambique. Overall, findings from our study align with the recommendations from a multinational DELPHI consensus to end COVID-19, which states that Governments should now prioritise early case detection so that health systems can facilitate earlier treatment and care.^24^

### Limitations

Despite this study providing the first qualitative assessment of decision-makers’ attitudes towards COVID-19 self-testing in Mozambique, their perspectives may not represent all possible opinions in the country. Additionally, the fact that the study was conducted when COVID-19 was not a public health concern could impact the responses given by DMs. However, this study can provide a current view of the importance of COVID-19 self-testing during the COVID-19 pandemic, given the reality of inadequate laboratory and health services access in crisis settings.

## CONCLUSIONS

Our study underscores the potential of COVID-19 self-testing as a critical tool in Mozambique, particularly in mitigating the strain on healthcare systems and facilitating timely diagnosis and treatment. The reflections of decision-makers reveal a consensus on the feasibility and acceptability of self-testing, highlighting key facilitators such as availability, ease of access, and integration with digital health systems. However, challenges such as cost, test accuracy, and referral processes must be addressed to ensure the effectiveness and scalability of this approach. Notably, the study emphasises the relevance of COVID-19 self-testing in humanitarian crises, which can play a pivotal role in controlling infection spread in vulnerable populations. These findings extend beyond Mozambique, offering valuable insights for other LMICs with similar healthcare constraints to manage other infectious diseases and allow self-care. By addressing shared challenges and leveraging comparable facilitators, LMICs can adopt and adapt self-testing strategies to strengthen healthcare delivery systems and improve health outcomes globally. This study underscores the importance of integrating self-testing into public health policies as part of a broader framework for infectious disease management, both during pandemics and in routine care. As Mozambique progresses, developing a comprehensive and inclusive COVID-19 testing policy, informed by lessons learned during the pandemic and supported by strong political will, it will be essential to realise the full benefits of self-testing. The alignment of our findings with global recommendations further reinforces the importance of early case detection and the strategic integration of self-testing within the broader public health framework (Figure).

**FIGURE.**
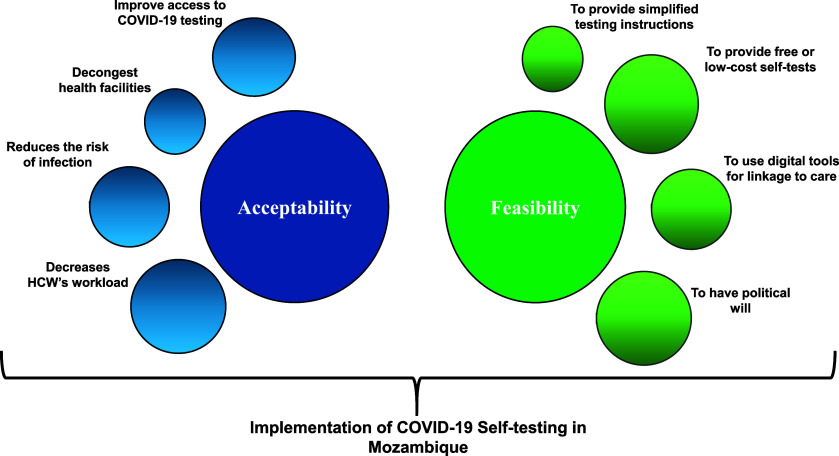
Results on the acceptability and feasibility of COVID-19 self-testing in Mozambique.
